# Selective Inhibition of Histone Deacetylase Class IIa With MC1568 Ameliorates Podocyte Injury

**DOI:** 10.3389/fmed.2022.848938

**Published:** 2022-04-14

**Authors:** Xu He, Tao Sun, Pei Zhang, Zhengkun Xia, Chunlin Gao, Hongqi Ren, Daxi Ji

**Affiliations:** ^1^Department of Pediatrics, Affiliated Jinling Hospital, Medical School of Nanjing University, Nanjing, China; ^2^Department of Pediatrics, Jinling Hospital, Nanjing, China; ^3^Department of Nephrology, Affiliated Huaihai Hospital of Xuzhou Medical University, Xuzhou, China; ^4^Department of Nephrology, BenQ Medical Center, The Affiliated BenQ Hospital of Nanjing Medical University, Nanjing, China

**Keywords:** podocyte injury, proteinuria, HDAC, β-catenin, nephrotic syndrome

## Abstract

Histone deacetylases (HDACs) inhibitors are promising therapeutic agents against proteinuric kidney diseases, here, we investigated the effect of MC1568, a selective inhibitor of HDAC class IIa, on the development and progression of nephrotic syndrome in a murine model induced by Adriamycin (ADR). In kidney tissues of FSGS patients, all four members of HDAC IIa were significantly upregulated in podocytes. In ADR-treated cultured human podocyte, expression of HDAC IIa were induced, meanwhile inhibition of HDAC IIa with MC1568 restored cytoskeleton structure and suppressed expression of desmin and α-SMA. In mice, administration of MC1568 at 14 days after ADR ameliorated proteinuria and podocyte injury, also decreased expression of Fibronectin and α-SMA. Mechanistically, MC1568 inhibited ADR induced β-catenin activation *in vitro* and *in vivo*. Together, these finding demonstrate that HDAC IIa inhibition ameliorates podocyte injury and proteinuria, which provide a possibility that MC1568 may be used in nephrotic syndrome.

## Introduction

Podocyte injury plays a crucial role in the pathogenesis of nephrotic syndrome (NS), which is one of the most common glomerular diseases and clinically characterized by severe proteinuria (1). Numerous studies have been done to identify the underlying molecular mechanism of podocyte injury during NS, which is valuable and helpful to explore and develop novel therapeutic chemicals by targeting podocytes (2), examples like GFB-887 (3), a small molecule selectively inhibiting TRPC5, has been demonstrated efficacy in a transgenic rat model of FSGS and hypertensive kidney disease, respectively, is now under a phase II clinical evaluation.

Histone deacetylases (HDACs) are involved in the development of chronic kidney disease, particularly in podocyte injury. Sirt1 and Sirt6 are NAD-dependent deacetylases. Sirt1 protects podocytes by maintaining PGC1-α expression *via* its deacetylase-activated transcriptional activity in mitochondria; decreased Sirt1 activity in podocytes results in podocyte apoptosis and proteinuria *via* acetylation of key transcriptional factors including FOXO4, p65, and STAT3 (4). Sirt6 has pleiotropic protective actions in podocytes, including anti-inflammatory and anti-apoptotic effects, while Sirt6 deficiency accelerates podocyte injury and proteinuria by targeting Notch signaling (5). HDAC4 contributes to podocyte injury by suppressing autophagy and exacerbating inflammation *via* STAT1 signaling in DN (6), mediates high glucose-induced podocyte apoptosis *via* upregulation of calcineurin (7). HDAC9 mediates podocyte injury by promoting reactive oxygen species (ROS) generation, cellular apoptosis, and inflammation *via* the JAK2/STAT3 pathway (8). HDAC1 and HDAC2 contributes to the pathogenesis of proteinuric kidney disease through the regulation of EGR1 in podocytes (9).

Pharmacological studies also suggested the therapeutic potential of HDAC inhibitors for podocyte injury and proteinuria. Valproic acid (VPA; a class I HDAC inhibitor) administration attenuated proteinuria and progression of renal disease whether before kidney injury or at the peak of proteinuria in Adriamycin (ADR)-induced NS in mice (10). VPA or SAHA (a pan-HDAC inhibitor) mitigated the degree of proteinuria and glomerulosclerosis in a genetically modified mice model of proteinuric kidney disease, doxycycline-induced Tln1*^fl/fl^* Pod-rtTA TetO-Cre mice (9).

Histone deacetylase Class IIa are larger than other HDACs, because HDAC IIa members contain nuclear export signals and can shuttle between the cytoplasm and the nucleus depending on their phosphorylation status. Evidences showed HDAC IIa inhibitors like TMP195 and MC1568 conferred protective effects in lipopolysaccharide (LPS)-induced acute kidney injury mice model (11) and unilateral ureteral obstruction (UUO) mice model (12), respectively, but whether HDAC IIa inhibitors prevent the development and progression of NS are still unknown. In this study, we examined MC1568 efficacy in ADR-induced NS mice model.

## Materials and Methods

### Patients

Patients (*n* = 5) with FSGS were diagnosed by renal biopsy at the Department of Nephrology, Nanjing BenQ Medical Center. The clinical characteristics of patients were detailed in Table 1. Healthy control glomerular samples (*n* = 3) were obtained from surgical nephrectomies.

**TABLE 1:** Clinical characteristics at the time of renal biopsy.

Gender	Age (years)	Serum creatinine (mg/dl)	24 h proteinuria (g/24 h)	Diagnosis
Female	28	0.52	0.86	FSGS
Male	32	0.67	2.14	FSGS
Male	29	0.46	2.12	FSGS
Male	19	0.36	1.65	FSGS
Female	42	0.89	2.77	FSGS

### Animal Experiments

Mice were grown and maintained following standard according to Jinling Hospital animal care guidelines, all experimental procedures were approved by the Institutional Animal Care and Use Committee at Jinling Hospital. Eight weeks-old male mice (C57BL/6N background) were subjected to 20 mg/kg ADR (MCE, HY-15142) injection *via* the tail vein. A total of 20 mg/kg MC1568 (Selleck, S1484) were intraperitoneal injected daily at 1 week after ADR injection for 3 weeks (1-week ADR plus 3 weeks MC1568), in total 4 weeks, mice were sacrificed. Urine albumin was quantified by Albuwell M ELISA kit (Exocell, 1011), Urine creatinine were examined with the same samples using Creatinine Companion (Exocell, 1012). The urine albumin excretion was assessed using the ratio of albumin to creatinine.

### Kidney Histology

Kidneys of mice were fixed with 4% paraformaldehyde (PFA) overnight at 4°C and embedded with paraffin. Paraffin-embedded kidney tissues were cut into 2-μm. Sections were stained with Periodic Acid Schiff (PAS) for histology analysis.

### Transmission Electron Microscopy

Kidney tissues of mice were fixed in 4% glutaraldehyde with 0.1 M cacodylate buffer (pH 7.2) for 4 h. Samples were further rinsed in cacodylate buffer and post-fixed in 1% OsO_4_ with 0.1 M cacodylate buffer (pH 7.4) for 24 h, dehydrated in a series of ethanol and infiltrated overnight in a 1:1 mixture of epoxy resin and propylene oxide. Specimens were embedded with Eponate 12 (Ted Pella, United States) and polymerized at 60°C for 24 h. Ultrathin sections were stained with lead citrate and uranyl acetate and viewed on a HITACHI H-7500 microscope.

### Immunohistochemistry Staining

Paraffin-embedded kidney sections from human and mouse were deparaffinized, blocked with 10% BSA in PBS for 30 min at room temperature (RT) and then incubated with primary antibodies at 4°C overnight. The next day, after several washes with PBS, incubate with secondary antibodies at RT for 1 h. For IHC staining, diaminobenzidine (DAB, Beyotime, China) color reaction was kept with a fixed exposure time for all experiments among the groups. Quantitation of IHC staining in the glomeruli was measured using ImageJ (NIH).

### Cell Culture, Treatment, Western Blotting, and Phalloidin Staining

The human podocyte cell line was provided by M. Saleem (University of Bristol, United Kingdom) and was cultured as described previously (13). Differentiated podocytes were treated with 10 μM ADR at indicated time points. MC1568 were treated 2 h before ADR administration with different dosages as indicated. Podocytes were lysed in RIPA buffer with protease inhibitors for following Western blotting (WB) analysis. Cell lysates were fractionated by SDS-PAGE and then transferred to polyvinylidene difluoride (PVDF) membranes (Millipore-Merck, United States). For phalloidin staining, podocyte were fixed in 4% PFA, permeabilized with PBS containing 0.1% Triton X-100 for 10 min at RT, then incubated with phalloidin-TRITC (MCE, HY-P2270).

### Antibodies

Histone deacetylase 4 (Proteintech, 17449-1-AP, WB, 1:1,000, IHC, 1:100), HDAC5 (Proteintech, 16166-1-AP, WB, 1:1,000, IHC, 1:100), HDAC7 (Santa Cruz, sc-74563, WB, 1:1,000, IHC, 1:100), HDAC9 (Santa Cruz, sc-398003, WB, 1:1,000, IHC, 1:100), Desmin (Proteintech, 16520-1-AP, WB, 1:1,000, IHC), α-SMA (Proteintech, 14395-1-AP, WB, 1:1,000, IHC, 1:200), Active β-catenin (CST, 8814, WB, 1:3,000, IHC, 1:200), GAPDH (Proteintech, 10494-1-AP, WB, 1:10,000), β-tubulin (Proteintech, 10094-1-AP, WB, 1:10,000).

### Statistics

Data were represented as mean ± SEM. Data between two groups were analyzed using *t*-tests. Data more than two groups were presented using two-way ANOVA with Bonferroni *post hoc* test. *p* < 0.05 were considered statistically significant.

## Results

### Histone Deacetylase IIa Members Are Upregulated in Podocyte From Patients With FSGS

**FIGURE 1:**
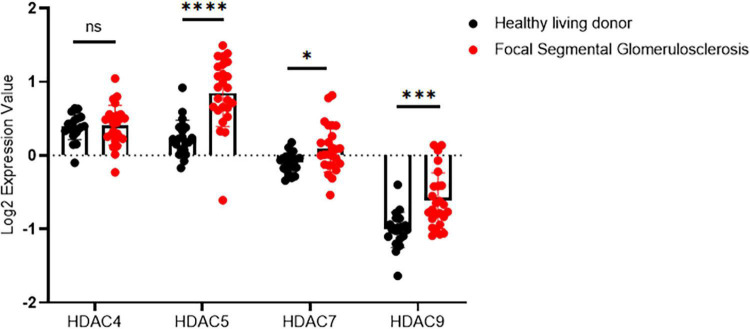
Glomerular expression of HDAC IIa in patients with FSGS using Nephroseq database. HDAC IIa members mRNA expression (median-centered Log_2_ expression value) in dissected glomeruli from healthy living donor (*n* = 21) and FSGS (*n* = 25). Statistical analysis was performed with two-tailed, unpaired *t*-test. *p < 0.05; ^***^p < 0.001; ^****^p < 0.0001.

**FIGURE 2:**
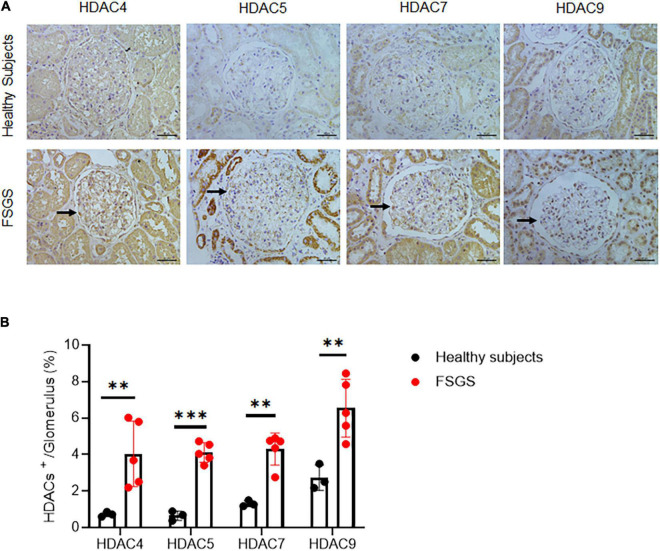
Validation of HDAC IIa in patients with FSGS. **(A)** IHC staining of HDAC IIa members (arrows indicate the positive stainings) in normal (*n* = 3) and FSGS (*n* = 5) kidney tissues. Scale bar, 40 μm. **(B)** Quantitative analysis of HDAC IIa IHC staining. Statistical analysis was performed with two-tailed, unpaired *t*-test. ^**^p < 0.01; ^***^p < 0.001.

We first examined the expression pattern of HDAC IIa in human kidneys using the Nephroseq database.^1^ In Ju CKD Glom (14), three members of HDAC IIa, including HDAC5, HDAC7, and HDAC9 but expect HDAC4, were all significantly upregulated in glomeruli in human FSGS compared with healthy controls (Figure 1). We next validated HDAC IIa expression in biopsy samples from FSGS patients, the IHC staining and quantifications (Figures 2A,B) showed glomerular expression of HDAC IIa were all marked increased, and HDAC IIa were predominantly expressed in podocytes. These data indicated HDAC IIa played roles during podocyte injury in FSGS.

### Inhibition Histone Deacetylase IIa With MC1568 Attenuates Adriamycin-Induced Podocyte Injury *in vitro*

**FIGURE 3:**
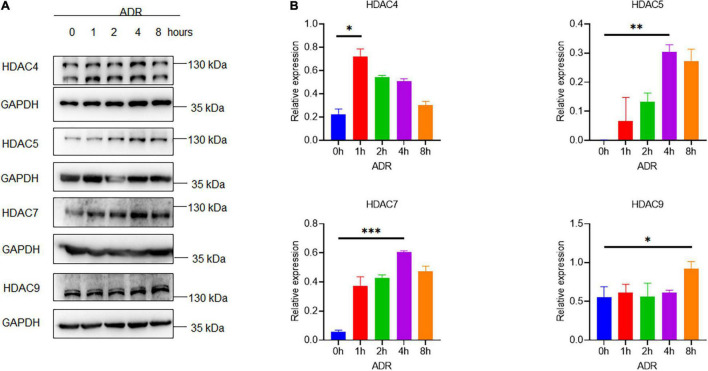
Histone deacetylase IIa expression in ADR-treated podocytes. **(A,B)** Representative Western blots with quantification for HDAC IIa members in ADR-treated podocytes statistical analysis was performed with two-way ANOVA. *p < 0.05; ^**^p < 0.01; ^***^p < 0.001.

**FIGURE 4:**
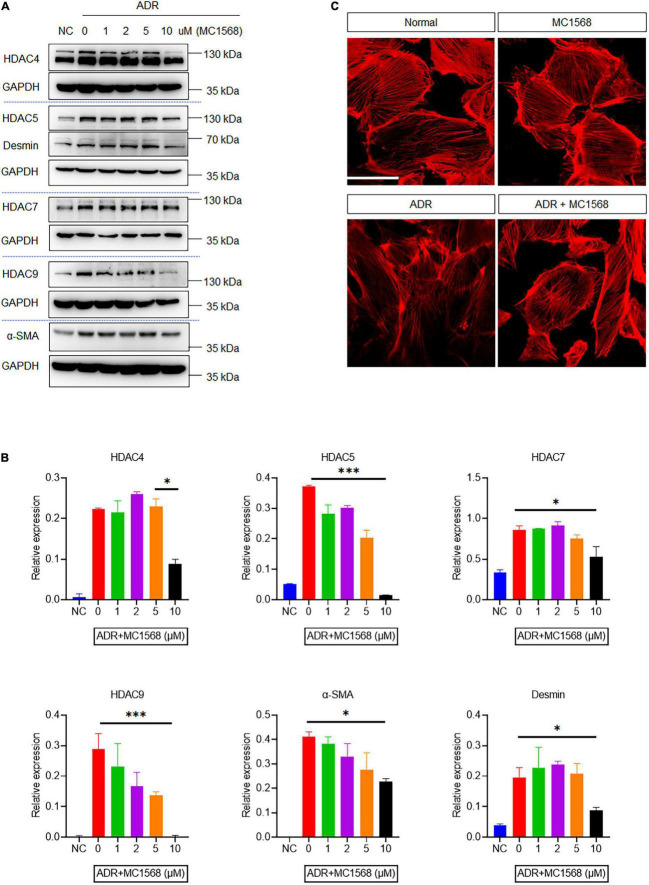
Histone deacetylase IIa inhibition with MC1568 protects podocyte from injury *in vitro*. **(A,B)** Immunoblotting and quantification analysis of HDAC IIa members, desmin and α-SMA in ADR-stimulated podocytes with or without MC1568 treatment. **(C)** Phalloidin staining in ADR-stimulated podocytes with or without MC1568 treatment. Scale bar, 50 μm. Statistical analysis was performed with two-way ANOVA. *p < 0.05; ^**^p < 0.01; ^***^p < 0.001.

To investigate the role of HDAC IIa in podocyte injury, we examined HDAC IIa members expression *in vitro* model of podocyte injury treated with ADR. After ADR treatment, WB results revealed that HDAC IIa members were increased time-dependently (Figure 3). To date, we blocked HDAC IIa expression with a selective inhibitor of HDAC IIa, MC1568, which was assessed in UUO mice model previously (12). In ADR-stimulated human podocytes, MC1568 pretreatment exhibited inhibitory effect dosage dependently, as shown in Figures 4A,B, 10 μm MC1568 was effective to suppress HDAC IIa expression induced by ADR. Podocyte epithelial-to-mesenchymal transition (EMT) as defined by desmin and α-SMA induction were also ameliorated by 10 μm MC1568. We also found ADR-induced cytoskeleton rearrangement and disruption were also restored by pretreatment with MC1568 (Figure 4C). Taken together, the *in vitro* results demonstrated the involvement of HDAC IIa in podocyte injury.

### Inhibition Histone Deacetylase IIa With MC1568 Attenuates Adriamycin-Induced Podocyte Injury and Proteinuria *in vivo*

**FIGURE 5:**
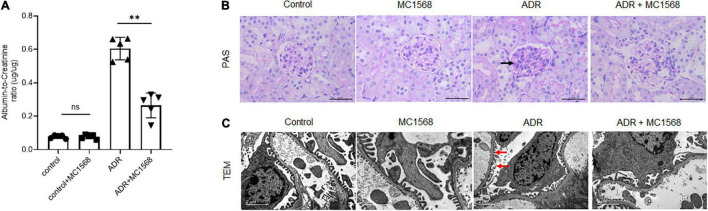
MC1568 administration ameliorates proteinuria and kidney function. **(A)** Urinary albumin/creatinine ratio in control and ADR-injected mice treated with or without MC1568. Statistical analysis was performed with two-way ANOVA. ^**^p < 0.01. **(B)** Representative PAS stained kidney images, black arrows indicate the glomerulosclerosis. Scale bar, 40 μm. **(C)** Representative transmission electron microscopy images, red arrows indicate the foot process effacement. Scale bar, 2 μm.

**FIGURE 6:**
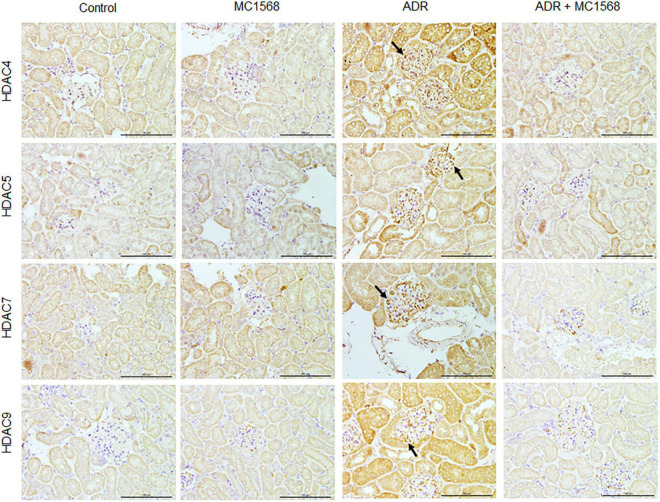
Inhibitory effects of MC1568 on HDAC IIa members. Representative images of immunohistochemical (IHC) of HDAC IIa members including HDAC4, HDAC5, HDAC7, and HDAC9 in control and ADR-injected mice treated with or without MC1568. Scale bar, 100 μm.

To assess the MC1568 inhibitory efficacy in podocyte injury and proteinuria *in vivo*, we treated mice with 20 mg/kg MC1568 daily at 1 week after ADR injection for 3 weeks. Much lower urine albumin-to-creatinine ratio (Figure 5A) were seen in ADR mice-treated with MC1568, compared with ADR mice. Kidney histology (Figure 5B) showed severe glomerulosclerosis and electron microscopy (EM) (Figure 5C) also displayed foot process effacement in ADR mice, compared with ADR mice-treated with MC1568. IHC staining demonstrated the *in vivo* inhibitory effects of MC1568 on HDAC IIa members (Figure 6). The data *in vivo* provided the direct evidence that MC1568 was effective to reduce podocyte injury and proteinuria.

### Activation of β-Catenin in Podocyte Injury Is Diminished by MC1568

**FIGURE 7:**
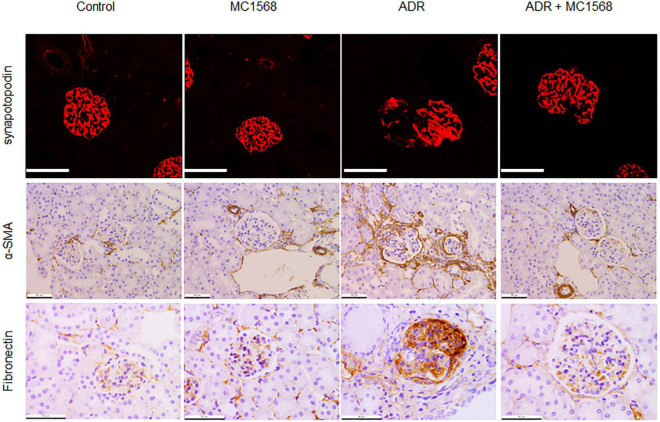
Inhibitory effects on podocyte markers. Representative images of immunohistochemical (IHC) and immunofluorescence (IF) staining of Synaptopodin, α-SMA, and Fibronectin in control and ADR-injected mice treated with or without MC1568. Scale bar, 50 μm.

**FIGURE 8:**
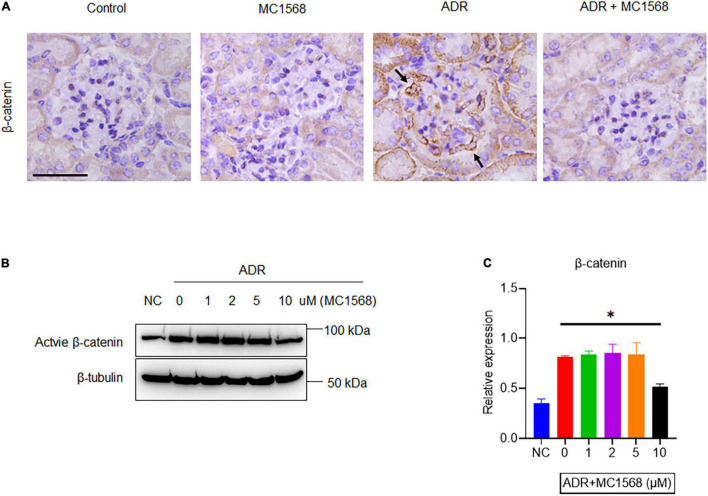
MC1568 suppresses ADR-induced β-catenin activation. **(A)** Representative images of IHC staining of β-catenin expression (arrows) in control and ADR-injected mice treated with or without MC1568. **(B,C)** Immunoblotting and quantification analysis of active β-catenin expression in ADR-stimulated podocytes with or without MC1568 treatment. Statistical analysis was performed with two-way ANOVA. *p < 0.05. Scale bar, 40 μm.

Similarly, IHC staining found decreased expression of Synaptopodin and increased glomerular expression of Fibronectin and α-SMA in ADR mice were downregulated by MC1568 administration (Figure 7). β-catenin activation plays central roles in podocyte injury and proteinuria, drives podocyte EMT (15). Thus, we investigated whether MC1568 could suppress β-catenin activation, which were examined *in vitro* and *in vivo*. IHC staining showed the glomerular segmental expression of β-catenin in ADR mice, but prohibited in ADR mice-treated with MC1568 (Figure 8A). In ADR-treated podocyte, 10 μm MC1568 markedly blocked activated β-catenin expression (Figures 8B,C). Collectively, MC1568 probably conferred protective effects by inhibiting β-catenin activation and EMT.

## Discussion

In the current study, we integrated transcriptional profiles from public database and IHC staining results in kidney tissues, identified HDAC IIa members were significantly upregulated in glomeruli of FSGS. We also found HDAC IIa members were markedly induced by ADR *in vitro* human podocytes. Administration of MC1568, a selective HDAC IIa inhibitor, attenuated ADR-induced podocyte injury *in vitro* and *in vivo*. Moreover, inhibition with MC1568 decreased ADR-induced β-catenin activation, fibronectin, desmin, and α-SMA expression.

The majority of patients with NS need to conceive daily treatment with corticosteroids until complete remission of proteinuria (16), side effects of corticosteroids are concerned, which promote us to explore potential drugs for alternative therapeutic intervention. HDAC inhibitors exhibited beneficial impacts against podocyte injury and proteinuria (17). HDAC IIa composes of HDAC4, HDAC5, HDAC7, and HDAC9, numbers of studies demonstrated HDAC IIa inhibition as an alternative approach to avoid the progression of various diseases, examples like breast tumors (18), pancreatic cancer (19), multiple myeloma (20), and type 2 diabetes (21). Recent evidences reveal that HDAC IIa inhibitor TMP195 alleviates LPS-induced acute kidney injury by reducing renal tubular cell apoptosis (11), another HDAC IIa inhibitor MC1568 inhibited development and progression of renal fibrosis in UUO mice model (12). However, therapeutic potential of HDAC IIa against podocyte injury and proteinuria is still unknown, here, we reported that HDAC IIa inhibitor MC1568 conferred therapeutic effect for podocyte injury and proteinuria.

Hyperactivation of β-catenin is sufficient to impair podocyte integrity and causes proteinuria, β-catenin is also a master transcription factor that controls the process of podocyte EMT (22). Activation of β-catenin is modulated by HDAC1 activity, in addition, they can form HDAC1/β-catenin complex to activate the expression of downstream target genes (23), thereby, whether HDAC IIa regulates β-catenin activation was asked. Both *in vitro* and *in vivo* results demonstrated that HDAC IIa inhibition blocked ADR-induced β-catenin activation, and its downstream desmin and α-SMA expression.

## Conclusion

In conclusion, HDAC IIa members are involved in podocyte injury, linking with β-catenin activation and EMT. Our findings provide the first evidence of therapeutic potential of MC1568 against podocyte injury and proteinuria.

## Data Availability Statement

The original contributions presented in the study are included in the article/supplementary material, further inquiries can be directed to the corresponding author/s.

## Ethics Statement

The studies involving human participants were reviewed and approved by the Human Subjects Committee of Jinling Hospital. The patients/participants provided their written informed consent to participate in this study. The animal study was reviewed and approved by the Institutional Animal Care and Use Committee at Jinling Hospital.

## Author Contributions

DJ conceived the research. DJ, HR, CG, and ZX designed the research. XH, TS, and PZ performed the experiments. XH wrote the manuscript. All authors have reviewed and approved the final version of the manuscript.

## Conflict of Interest

The authors declare that the research was conducted in the absence of any commercial or financial relationships that could be construed as a potential conflict of interest.

## Publisher’s Note

All claims expressed in this article are solely those of the authors and do not necessarily represent those of their affiliated organizations, or those of the publisher, the editors and the reviewers. Any product that may be evaluated in this article, or claim that may be made by its manufacturer, is not guaranteed or endorsed by the publisher.

## Footnotes

1
www.nephroseq.org
